# General and subspecialist pediatrician perspectives on barriers and strategies for referral: a latent profile analysis

**DOI:** 10.1186/s12887-023-04400-8

**Published:** 2023-11-18

**Authors:** James C. Bohnhoff, Katherine Guyon-Harris, Kelsey Schweiberger, Kristin N. Ray

**Affiliations:** 1 Department of Pediatrics, Maine Health, 1577 Congress St Fl 1, Portland, ME 04102 USA; 2 Center for Interdisciplinary Population and Health Research, Maine Health Institute of Research, Scarborough, ME USA; 3 Department of Pediatrics, Tufts University School of Medicine, Boston, MA USA; 4 Department of Pediatrics, University of Pittsburgh School of Medicine, Pittsburgh, PA USA; 5 Division of General Academic Pediatrics, UPMC Childrens Hospital of Pittsburgh, Pittsburgh, PA USA

**Keywords:** Access, Pediatric Subspecialty, Telemedicine, Workforce, Scheduling

## Abstract

**Background:**

Children in need of pediatric subspecialty care may encounter multiple barriers, and multiple strategies have been suggested to improve access. The purpose of this study was to describe the perceptions of general and subspecialty pediatric physicians regarding barriers to subspecialty care and the value of strategies to improve subspecialty access.

**Methods:**

We surveyed a national sample of 1680 general pediatricians and pediatric subspecialists in May and June 2020 regarding 11 barriers to subspecialty care and 9 strategies to improve access to subspecialty care, selected from recent literature. Using latent profile analysis, respondents were grouped according to the degree to which they believed each of the barriers impacted access to subspecialty care. Using chi-squared tests, we compared the profiles based on respondent characteristics and perspectives on strategies to improve access.

**Results:**

The response rate was 17%. In 263 responses completed and eligible for inclusion, the barriers most frequently described as “major problems” were wait times (57%), lack of subspecialists (45%) and difficulty scheduling (41%). Respondents were classified into 4 profiles: “Broad concerns,” “Subspecialist availability concerns,” “Clinician communication concerns,” and “Few concerns.” These profiles varied significantly by respondent specialty (*p* < .001, with medical subspecialists overrepresented in the “Clinician communication” profile, psychiatrists in the “subspecialist availability” profile, and surgeons in the “few concerns” profile); and by respondents’ typical wait time for appointments (*p* < .001, with physicians with the longest wait times overrepresented in the “subspecialist availability” profile).

**Conclusions:**

We found specific profiles in clinician views regarding barriers to subspecialty care which were associated with perspectives on strategies aimed at overcoming these barriers. These results suggest that health systems aiming to improve subspecialty access should first identify the barriers and preferences specific to local clinicians.

## Background

In the United States, on average, one subspecialty visit occurs for each child per year [1], often at the direction of a primary care provider (PCP). Unfortunately, many referrals result in a significantly delayed subspecialty appointment [2], an unattended, scheduled appointment, or no scheduled appointment at all [3, 4], while some completed appointments are deemed by subspecialists to be unnecessary [5–7]. PCPs [8–10], families [11], and subspecialists [9, 12] alike describe the referral process as ineffective and inefficient, and the breakdown of referrals can threaten patient safety by contributing to delays in diagnoses and treatment [13]. Additionally, patients who do not or cannot access subspecialty care may experience decreased quality of life and increased need for acute care, which may also increase healthcare costs [14, 15].

The complexity of the referral process makes it difficult to assess and improve. Referrals may be impeded by multiple barriers including travel barriers, subspecialist shortages, and communication barriers between referrers, subspecialists, and families [11, 16]. In a 2010 national survey the general pediatrician respondents perceived long wait times, low subspecialist availability, and low acceptance of uninsured patients to be the greatest barriers to subspecialist referral [10]. Less is known, however, about the barriers perceived by subspecialists receiving referrals from pediatricians. In addition, although multiple strategies such as telemedicine, e-consultation [2, 17], the inclusion of generalist pediatricians and/or advance practice providers (APPs) into specialty teams [18–20], and referral guidelines [21] have been designed to improve the referral process, implementation has varied widely [22, 23]. Appropriate implementation of these strategies will depend on identifying the most problematic referral barriers and choosing referral strategies that are well-suited to addressing these barriers [24] and acceptable to all those involved in referrals, including families, referring clinicians, and subspecialists. To date, very little research has compared the actual or perceived effectiveness of different referral strategies or assessed how strategies might pair with specific situational barriers. We address these knowledge gaps with results from a national sample of pediatricians and pediatric subspecialists, assessing respondents’ views regarding barriers to subspecialty care, referral strategies and the associations between their perspectives on these topics using latent profile analysis.

## Methods

We analyzed results from a cross-sectional survey administered electronically and by postal mail to a national sample of general pediatricians and pediatric subspecialists, which included multiple topics related to subspecialty referral and telemedicine. Other results from this survey, along with all survey questions, have been reported elsewhere [22, 25]; we focus here on survey questions relating to barriers and potential solutions for subspecialty referral. This survey was administered through postal mail and follow-up email to a random sample of 1680 general pediatricians and medical, surgical, and psychiatric pediatric subspecialists between May and June 2020. Potential respondents were identified from the American Medical Association Masterfile accessed through third-party vendor DMD Marketing Corp (Rosemont, IL), which selected from a list of providers to equally represent general pediatricians and pediatric subspecialists in the four United States census regions. After completing the survey, participants could register for a chance to receive one of thirty-two $100 gift cards. Data from paper surveys were manually entered and double checked by two study members to minimize errors.

Participants were asked to rate 11 potential barriers to subspecialty care as posing a “major problem”, “minor problem,” or “not a problem,” and to rate 9 potential access strategies on a 5-point Likert scale ranging from likely to “significantly worsen” to “significantly improve” access to high-quality subspecialty care. These barriers and strategies were informed by the existing literature on pediatric subspecialty access [3, 10, 24]. Respondents also reported information on their demographics and practice setting. The survey was reviewed by members of the Supporting Pediatric Research on Outcomes and Utilization of Telehealth (SPROUT) collaborative group and was completed through an electronic survey tool (Qualtrics XM, Provo, Utah).

Respondents were eligible to participate if they were general pediatricians, pediatric medical or surgical subspecialists, or child psychiatrists who had completed medical training, provided medical care at least one day per week, and were in the US while completing the survey. We also excluded responses in which the questions assessing barriers to care were not completed. We then utilized Latent Profile Analysis (LPA), a technique which identifies groups (or profiles) of respondents with similarities across multiple variables In this analysis, LPA provided a rigorous methodology with which we could identify groups (or profiles) of respondents based not on one variable but on similar patterns of responses across the 11 survey questions assessing different barriers to subspecialty care (which we reduced to binary variables denoting whether a barrier was perceived to be a “major problem”). The model fit (the degree to which respondents within profiles answered similarly regarding barriers, and to which respondents between profiles answered differently) and classification quality (the frequency with which an individual would be sorted into the appropriate profile) were evaluated for profile solutions with anywhere from 2 to 5 profiles using previously published and empirically supported measures including fit indices, indices of model classification quality, and interpretability [26, 27]. After selecting the best fitting model, we used chi-squared analyses to assess differences between the profiles in terms of clinical features (specialty, years in clinical practice, practice setting, whether respondents’ salaries were primarily determined by relative value units (RVUs), and a typical wait time for nonemergent appointments). We also used chi-squared analysis to assess differences by profile in perceived value of access strategies, with responses reduced into binary variables (perceived worsening or no effect on access vs perceived improvement). LPA was performed using Mplus version 8 (Muthén & Muthén, Los Angeles, California); profile membership was extracted into SPSS (version 23), where chi-square analyses were completed.

## Results

Of 1680 surveys distributed, 98 were returned as undeliverable, and we received 301 responses. Because 16 surveys were not eligible for inclusion and 22 respondents did not answer all questions needed for analysis, 263 surveys were analyzed (263/1582 = 17% response rate). These respondents included general pediatricians (*n* = 116) and pediatric medical (*n* = 85), psychiatric (*n* = 42), and surgical (*n* = 20) subspecialists. The analyzed respondents practiced medicine in 39 different states, and 96% practiced in locations classified as “metropolitan” based on their Rural–Urban Commuting Area codes [28]. As reported elsewhere, respondent characteristics were similar to the population sampled [18]. In the overall population, the barriers most often perceived to be major problems were long wait times (57%), lack of subspecialists (45%) and difficulty scheduling (41%).

**Fig. 1:**
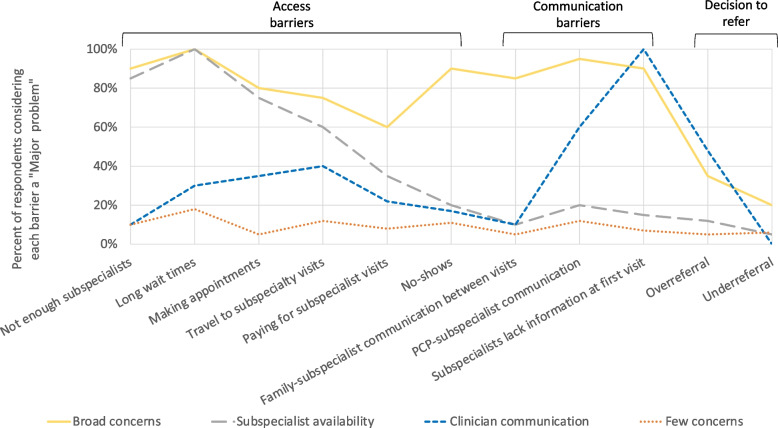
Perceived impact of barriers to subspecialty care by profile

The 4-profile solution was chosen as the best fitting model based on a combination of fit indices, classification quality, and interpretability of the profiles (Table 1). We describe these barrier perception profiles as “Broad concerns” (*n* = 30, 11%) “Subspecialist availability concerns” (*n* = 96, 36%), “Clinician communication concerns” (*n* = 23, 9%,), and “Few concerns” (*n* = 115, 44%). Figure 1 illustrates the perceptions around barriers to subspecialty care for each profile.

**Table 1:** Latent profile models for perceived barriers to pediatric subspecialty care

	AIC	SSA BIC	LMR-LRT	*p*-value	BLRT	*p*-value	Entropy	Classification probabilities
2-profile	2838.052	2847.377	-1597.26	< .0001	-1597.26	< .0001	0.833	.945-.964
3-profile	2772.165	2786.356	-1396.03	0.3523	-1396.03	< .0001	0.835	.865-.930
4-profile	2756.176	2775.232	-1351.08	0.2633	-1351.08	< .0001	0.848	.873-.915
5-profile	2750.719	2774.64	-1331.09	0.3652	-1331.09	0.1053	0.787	.689-.959

The collective interpretation of multiple fit indices and indices of classification quality are used to guide the selection of the most appropriate multi-profile model. Interpretation of each fit index has been summarized previously [26]. *AIC* Akaike Information Criteria, *SSA BIC* sample size adjusted Bayesian Information Criteria, *LMR-LRT* Lo-Mendell-Rubin Likelihood Ratio Test, *BLRT* Bootstrap Likelihood Ratio Test. AIC, SSA BIC, LMR-LRT and BLRT are fit indices. Entropy and Classification probabilities are indices of classification quality

The four profiles of respondents varied significantly in their specialties (*p* < 0.001, Table 2). Medical subspecialists were more frequently categorized as concerned with “Clinician communication” (14%, vs 9% of all respondents) and less frequently categorized as concerned with “Subspecialist availability” (31%, vs 36% of all respondents). Psychiatrists were more frequently categorized as having “Broad concerns” (19%, vs 11% of all respondents) and “Subspecialist availability concerns” (71%, vs 36% of all respondents) profiles, and surgical subspecialists were more likely categorized as “Few concerns” (85%, vs 44% of all respondents). The profiles were not associated with academic practice setting or respondents’ years in practice but were associated with the average time respondents perceived patients waited for a new visit to their clinic, with individuals reporting the longest wait times more frequently falling into “Broad concerns” or “Subspecialist availability concerns” profiles (*p* < 0.001).

**Table 2:** Practice characteristics, by profile

	Broad concerns	Subspecialist availability	Clinician communication	Few concerns	P
All respondents (*n* = 263)	30 (11%)	95 (36%)	23 (9%)	115 (44%)	
**Specialty**					** < .001**
**General pediatricians (*n* = 116)**	**12 (10%)**	**38 (33%)**	**10 (9%)**	**56 (48%)**	
**Medical subspecialists (*n* = 85)**	**9 (11%)**	**26 (31%)**	**12 (14%)**	**38 (45%)**	
**Psychiatrists (*n* = 42)**	**8 (19%)**	**30 (71%)**	**0 (0%)**	**4 (10%)**	
**Surgeons (*n* = 20)**	**1 (5%)**	**1 (5%)**	**1 (5%)**	**17 (85%)**	
Years of clinical practice					.644
0–5 (*n* = 30)	4 (13%)	12 (40%)	3 (10%)	11 (37%)	
6–10 (*n* = 35)	4 (11%)	9 (26%)	4 (11%)	18 (51%)	
11–20 (*n* = 76)	4 (5%)	31 (41%)	6 (8%)	35 (46%)	
> 20 (*n* = 122)	18 (15%)	43 (35%)	10 (8%)	51 (42%)	
Academic Practice Setting – Yes (*n* = 105)	13 (12%)	36 (34%)	15 (14%)	41 (39%)	.062
Percent clinical time					.066
< 60% (*n* = 32)	4 (13%)	13 (41%)	12 (6%)	13 (41%)	
60–79% (*n* = 43)	4 (9%)	17 (40%)	9 (21%)	13 (30%)	
≥ 80% (*n* = 188)	22 (12%)	65 (35%)	12 (6%)	89 (47%)	
Paid through RVUs – Yes (*n* = 89)	8 (9%)	31 (35%)	7 (8%)	43 (48%)	.722
**Typical wait time (in days)**					** < .001**
** < 8 (*n* = 114)**	**15 (13%)**	**30 (26%)**	**6 (5%)**	**63 (55%)**	
**8–14 (*n* = 50)**	**2 (4%)**	**17 (34%)**	**7 (14%)**	**24 (48%)**	
**15–28 (*n* = 45)**	**6 (13%)**	**16 (36%)**	**6 (13%)**	**17 (38%)**	
**29–60 (*n* = 28)**	**4 (14%)**	**15 (54%)**	**1 (4%)**	**8 (29%)**	
** > 60 (*n* = 19)**	**3 (16%)**	**15 (79%)**	**0 (0%)**	**1 (5%)**	

*RVUs* relative value units. For percent clinical time, responses of < 20%, 20–39%, and 40–59% were combined to avoid unacceptably low cell size. For typical wait time, 7 nonrespondents are not included in counts. *P* values evaluate Chi-squared tests for variations in practice characteristics among profiles

The access strategies felt to be beneficial by the largest number of respondents were telemedicine (85%), referral hotlines (81%), and more subspecialists (80%, Table 3). In contrast, 61% of respondents felt that increasing the number of nurse practitioners and physician assistants would be beneficial to access. Respondents varied significantly by barrier perception profile in their views on telemedicine (*p* = 0.049) and increasing the numbers of subspecialists (*p* < 0.001); for both of these strategies, respondents in the “Subspecialist availability concerns” profile were most likely to anticipate a benefit, and respondents in the “Clinician communication concerns” profile were the least likely to anticipate a benefit.

**Table 3:** Percent of respondents anticipating benefit of access strategies, by profile

	All respondents (*n* = 263)	Broad Concerns (*n* = 30)	Subspecialist availability (*n* = 95)	Clinician communication (*n* = 23)	Few Concerns (*n* = 115)	P
**Telemedicine**	**225 (85%)**	**27 (90%)**	**87 (92%)**	**16 (70%)**	**95 (83%)**	**0.049**
Referral Hotlines	214 (81%)	24 (80%)	79 (83%)	17 (74%)	94 (82%)	0.821
**More specialists**	**210 (80%)**	**29 (97%)**	**89 (94%)**	**14 (61%)**	**78 (68%)**	** < .001**
Scheduling Improvements	205 (78%)	27 (90%)	71 (75%)	19 (83%)	88 (77%)	0.284
Training for PCPs	199 (75%)	23 (77%)	74 (78%)	16 (70%)	86 (75%)	0.892
Store-and Forward	194 (74%)	24 (80%)	69 (73%)	14 (61%)	88 (77%)	0.36
Referral Guidelines	192 (73%)	20 (67%)	73 (77%)	17 (74%)	82 (71%)	0.748
Portal Communications	189 (72%)	22 (73%)	63 (66%)	14 (61%)	90 (78%)	0.136
More nurse practitioners and physician assistants	162 (61%)	20 (67%)	65 (68%)	12 (52%)	65 (57%)	0.268

*PCPs* primary care physicians. *P* values evaluate Chi-squared tests for whether perspectives on each strategy vary by profile

## Discussion

In this national sample of general pediatricians and pediatric subspecialists, respondents reported that the most significant barriers to high-quality subspecialty care were related to availability of subspecialists and subspecialty appointments: long wait times, lack of subspecialists, and difficulty scheduling. Similarly, respondents were most optimistic about strategies aimed at increasing the availability of subspecialists and subspecialty knowledge: telemedicine, referral hotlines, and greater numbers of subspecialists. Despite these general trends, however, latent profile analysis revealed four distinct groups with different concerns. The largest group had relatively “Few concerns” for all the studied barriers. Surgeons were overrepresented in this group, which may relate to other work showing that patients referred to surgical specialists may face less barriers than those referred to medical care [3]. The second largest group expressed concern most frequently for the supply of subspecialists (85%) and the related concern of long wait times (100%). Psychiatrists were overrepresented in this group, consistent with known concerns about the mental health workforce [29, 30]. A smaller group, by contrast, reported low concern for workforce supply and initial access barriers, but higher concern for “Clinician communication” barriers: subspecialists lacking information when first encountering patients (100%) and PCP-subspecialist communication (60%). This group also had the highest level of concern (48%) regarding over-referral. The existence of this “Clinician communication concerns” profile, which was composed primarily of general pediatricians and medical subspecialists, suggests the heterogeneity of barriers to high-quality pediatric subspecialty care, and the importance of defining these barriers and implementing strategies that overcome the specific barriers affecting a given institution, subspecialty, and patient population. This general principle – that referral strategies should be matched with specific clinical settings, should be considered by health systems, health systems researchers, and payers and policymakers interested in improving child access and health outcomes through improved referrals. Since we did not find that respondents in the “Clinician communication concerns” profile were significantly more likely to endorse any of the strategies we proposed, more research may be necessary to identify strategies appropriate for situations where over-referral and gaps in communication are of concern.

This work illustrates two major strengths of latent profile analysis. First, grouping respondents into profiles allowed us to identify heterogeneity among our respondents that extended beyond their answers to any one individual question. We were also able to describe the associations between individuals’ responses to different questions; for example, describing the connections between respondents’ views on barriers and potential solutions, without listing out the associations between views on 9 barriers and 11 referral strategies). However, this study also had limitations: our response rate was only 17%, and although this is a similar rate to other surveys of physicians [31, 32] and the characteristics of the respondents did not differ greatly from the national random sample in measured characteristics (respondent specialty, gender, years in practice, and census region) [22], they may have differed in some unmeasured characteristics which would have influenced their responses. We also chose to analyze responses from generalists and subspecialists together. This allowed us to note differences in concerns between referral placers and receivers, and to identify barrier concern profiles that included both groups, but may decrease the sensitivity of our results since, for example, subspecialists answered questions about referral barriers only within their own specialty, but generalists were likely considering referrals overall. The third major participant in subspecialty referral, patients and their families, were not included in this survey. Because we asked only about specific access strategies and did not solicit free-response perspectives on access strategies, our conclusions are constrained to the barriers and solutions about which we explicitly asked. We cannot address, for example, the inclusion of pediatric generalists into subspecialty teams. Finally, this study was carried out within the first six months of the coronavirus-2019 pandemic in the United States. This pandemic affected clinical practice in multiple ways, including increasing telemedicine use and decreasing demand for many pediatric subspecialties. These changes may have influenced our survey responses, and it remains to be seen to what degree these pandemic-related shifts will persist.

## Conclusions

We found that although many pediatric generalists and subspecialists expressed concerns related to patients’ ability to access subspecialists, this concern was not universal, with some physicians alternatively concerned with over-referral, PCP-subspecialist communication, and information-sharing. Systems hoping to improve access to subspecialty pediatric care should avoid a one-size-fits-all approach and should instead match access strategies to the barriers experienced in specific clinical settings and by specific patient populations.

## Data Availability

The datasets used and/or analyzed during the current study available from the corresponding author on reasonable request.

## Abbreviations

AIC: Akaike Information Criteria
BLRT: Bootstrap Likelihood Ratio Test
LPA: Latent profile analysis
PCP: Primary care provider
RVU: Relative value unit
SSA BIC: Sample size adjusted Bayesian Information Criteria
